# Modulation of the behavioral and electrical responses to the repellent DEET elicited by the pre-exposure to the same compound in *Blattella germanica*

**DOI:** 10.7717/peerj.2150

**Published:** 2016-06-28

**Authors:** Valeria Sfara, Gastón A. Mougabure-Cueto, Paola A. González-Audino

**Affiliations:** 1 Instituto de Investigación e Ingeniería Ambiental (3iA), Universidad Nacional de San Martín (UNSAM), San Martín, Buenos Aires, Argentina; 2 Centro de Referencia de Vectores (CeReVe), Ministerio de Salud de la Nación, Santa María de Punilla, Córdoba, Argentina; 3 Centro de Investigaciones de Plagas e Insecticidas (CIPEIN-UNIDEF-CONICET), Buenos Aires, Argentina

**Keywords:** *Blattella germanica*, Adaptation, DEET, Repellency

## Abstract

Insects under different stimuli from the environment modify behavioural responses due to changes in the sensitivity of neurons at the peripheral and/or at the central level of the nervous system. This phenomenon is called neuronal plasticity, and sensory adaptation is an example of it. An insect repellent is a chemical that produces oriented movements of the insects away from its source. In this work we studied the modulation of the behavioural and electrical response to the repellent N, N-diethyl-3-methylbenzamide (DEET) in males of the German cockroach *B. germanica* produced by previous exposure to the same repellent.

**Methods.** We determined repellency using a circular arena, one half of which was treated with DEET. The time spent by insects in each half of the arena was measured, and a repellency coefficient (RC) was calculated. The RCs of pre-exposed and non-pre-exposed insects were compared. To determine a possible role of nitric oxide in the modulation of the response to DEET after pre-exposure, the nitric oxide donor S-nitroso-acetyl-cysteine (SNAC) was applied on cockroaches’ antennae. The electrical activity of the cockroaches’ antennae in response to DEET was recorded using electroantennogram (EAG) technique. The response to DEET was recorded also after a long stimulation with the same repellent, and after topical application of SNAC and dbcGMP (a cGMP analogue) on the antennae.

**Results.** We found that previous exposure of *B. germanica* males to the repellent DEET produced an increase of the repellency at the behavioural level, measured as RC. A possible role of nitric oxide (NO) in the transduction pathway of this phenomenon is suggested, since treatment of the cockroaches with the NO donor SNAC also produced an increase of the repellency elicited by DEET. On the other hand, the response of the cockroaches’ antennae exposed to DEET was determined electrophysiologically. The electrical activity in response to DEET decreased when the insects’ antennae were stimulated with a long pulse of the repellent. The activity of the antennae was restored after 10 min. Treatment of the antennae either with SNAC or dbGMPc also produced a decrease in the response of the antennae to the repellent.

**Discussion.**The previous exposure to a chemical stimulus can modify the behaviour associated to the same stimulus, increasing or decreasing the behavioural response. In the case of DEET we found that pre-exposure increased DEET repellency in male cockroaches. We also found NO involvement in a similar phenomenon. On the other hand, the test showed that DEET is perceived by insects’ antennae as an odour. A long exposure of the antennae to DEET caused a transient decrease in the response of the antennae to the same compound. The same effect was achieved by treating the antennae with SNAC or dbcGMP, suggesting the involvement of the NO/cGMP system in the transduction pathway of the sensory adaptation phenomenon elicited by an odour in this species.

## Introduction

An insect repellent has been defined as a chemical substance that produces oriented movements of the insects away from its source (Dethier, Browne & Smith, 1960; Barton Browne, 1977). N, N-diethyl-3-methylbenzamide (DEET) is an insect repellent used worldwide. The repellent properties of DEET were discovered in 1946. Ten years later, it was available to consumers and became a successful product due to its effectiveness, persistence, and low human toxicity (Frances, 2007). The effect of DEET has been proved in several species, including haematophagous and non-haematophagous insects (Alzogaray, Fontán & Zerba, 2000; Reeder et al., 2001; Klun, Khrimiana & Debboun, 2006; Sfara, Zerba & Alzogaray, 2006; Sfara, Zerba & Alzogaray, 2008).

In nature, the exposure of insects to different stimuli produces changes in the behavioural response to repeated stimulation, allowing the animal to be more efficient. These changes are associated with variations in the sensitivity of neurons at the peripheral as well as at the central level. This phenomenon is known as neuronal plasticity. Sensory adaptation is an example of neuronal plasticity that occurs in almost all sensory neurons, and consists of the regulation of the sensitivity of the sensory system to different stimulus intensities (Zufall & Leinders-Zufall, 2000; Kleineidam et al., 2000). These phenomena are usually transient and do not imply permanent changes at the cellular level, such as protein synthesis.

Electrophysiological studies performed in different mosquitoes species showed that DEET acts as a sensory stimulus and can be detected by the antennae (Davis & Rebert, 1972; Ditzen, Pellegrino & Vosshall, 2008; Syed & Leal, 2008; Bohbot & Dickens, 2010; Lee, Kim & Montell, 2010; Pellegrino et al., 2011; De Gennaro et al., 2013; Sanford, Shields & Dickens, 2013; Stanczyk et al., 2013). In these studies, authors could identify the receptors that respond to DEET in *Aedes* and *Culex sp.* by means of single sensillum recording.

Behavioural experiments performed in mosquitoes and Triatominae bugs showed that the responsiveness to DEET can change due to experience. Nymphs of the hemipteran *Rhodnius prolixus* are less repelled by DEET after previous exposure to the same substance (Sfara et al., 2011). In this case, lack of repellency was observed after an exposure of at least 10 min, and the response was totally restored after 20 min. A similar result was obtained in *Aedes aegypti* mosquitoes (Stanczyk et al., 2013).

Regarding the transduction pathway of sensory adaptation in olfactory neurons, treatments with the nitric oxide (NO) donor S-nitroso-acetyl-L-cysteine (SNAC) and the cGMP analogue, dibutyryl cyclic GMP (dbcGMP), produced a decrease in the repellency elicited by DEET in nymphs of *R. prolixus* (Sfara, Zerba & Alzogaray, 2008), suggesting a possible role of the NO/cGMP system in the transduction pathway of this phenomenon.

Although DEET has been used for several decades as the active principle on most of the repellents available in the market for personal protection against the biting of haematophagous insects, its effectiveness has been proved also in non-haematophagous species, such as *Drosophila melanogaster* and *B. germanica* (Reeder et al., 2001; Sfara et al., 2013). Besides their use for personal protection, repellent formulations may be used for spatial applications to avoid the access of insects to certain areas. In the case of cockroaches, repellency caused by DEET may be a useful tool for spatial exclusion of the insects. This strategy may help to reduce the use of conventional insecticides in programs of integrated pest management, for cockroaches’ control. A repellent substance acts as an excito-repellent, when it is applied on the skin of the host and the substance increases the locomotor activity of the insect (Alipour et al., 2013; Diotaiuti et al., 2000). As a consequence of this, insects move against the source of the repellent, i.e., the host skin. On the other side, an insect repellent can be considered a spatial repellent if it is applied on a surface, in order to exclude insects from a certain area.

To test the hypothesis that DEET is an odour molecule acting as a repellent in non-haematophagous insects, in this work we studied the effect of the pre-exposure in the response elicited by the insect repellent DEET at the behavioural and electrophysiological levels, and a possible role of the NO/cGMP pathway in such action, in the German cockroach *Blattella germanica.*

## Materials and Methods

### Biological material

Adult males of *B.germanica* of 7–20 days-old since molt were used in the experiments. Insects were reared in the laboratory in an environmental chamber, maintained at 25°C and 12:12 Light:Dark photoperiod. Inside the chamber, cockroaches’ colonies were maintained in plastic containers (20 × 20 × 20 cm) in which all stadia were reared together, with rat pellet as food source and water *ad libitum*. Fifth instar nymphs were then separated in circular plastic containers (15 cm diameter; 20 cm height), and were maintained also with food and water *ad libitum* until molt. After the emergence of adults males were separated for their experimental use.

### Chemicals

N, N-diethyl-3-methylbenzamide (DEET 97% pure) was from Aldrich (Milwaukee, WI, USA). Acetone was analytical grade 99% pure was from Merck (Darmstadt, Germany).

The NO donor S-Nitroso-acetyl-cysteine (SNAC) was synthesized in our laboratory by acid-catalyzed nitrosation of acetyl-L-cysteine as described by Mathews & Kerr (1993). Briefly, 32.6 mg of N-acetyl-L-cysteine was weighed in a vial of 4 ml and dissolved in 250 µl of distilled water (Solution A). Similarly, 46 mg of NaNO_2_ were dissolved in 500 µl of a 0.1% EDTA solution (Solution B). Then, 150 µl of Solution B was gently added to Solution A. These compounds react immediately, and the resulting solution becomes red. This red solution was acidified to pH = 2 by adding HCl 1N and then left to stand at room temperature for 5 min. The solution was then neutralized to pH = 7 with NaOH 0.5 N and taken to a final volume of 5 ml with water with Triton X-100 1%, obtaining a 40 mM solution of SNAC. Triton X-100 was used to facilitate posterior penetration through insect’s cuticle during topical application. The chemical identity and the stability of SNAC solution were proved by spectrophotometry at 330 nm, based on the molar extinction coefficient of 727 (Mathews & Kerr, 1993). SNAC was freshly prepared daily.

Dibutyryl cyclic GMP (dbcGMP) was purchased from Sigma-Aldrich (Milwaukee, WI, USA).

### Behavioural bioassays

The effect of previous exposure to DEET in the response of the insects to the same substance was determined at the behavioural level as follows.

An experimental arena constituted by a circular transparent plastic container of 9 cm in diameter and 4 cm height provided with a lid, was used to pre-expose the insects. A circular piece of Whatman No. 1 filter paper of the same diameter (Whatman International Ltd., Maidstone, UK) was treated with 0.5 ml of a solution of 250 mg/ml of DEET in acetone (obtaining a concentration of 2 mg/cm^2^ on the surface). After solvent evaporation, the filter paper was placed in the base of the container. One insect was gently placed inside of the container that was then closed. The insect walked on a piece of gauze placed on the experimental arena floor, which was separated a few millimeters from the DEET-treated filter paper. In this way, the insects were exposed only to DEET vapours, but were not in contact with the treated surface. Insect was kept inside the containers for 20 min. Immediately after, repellency elicited by 100 mg/ml of DEET (700 µg/cm^2^ on the surface) was determined. Control groups were pre-exposed to filter papers treated only with acetone. As an additional control, repellency elicited by 100 mg/ml of DEET was calculated in non-treated insects.

Repellency was determined using a “spatial exclusion protocol”: a circular piece of Whatman No. 1 filter paper (diameter: 11 cm) was cut into halves (Zone I and Zone II). Zone I was treated with 0.35 ml of acetone, whereas Zone II was treated with 0.35 ml of a solution of DEET in acetone (100 mg/ml). After solvent evaporation, both filter paper pieces were fitted together and located on the test arena floor. A glass ring (height: 4.5 cm; diameter: 9 cm) was used to prevent the insect from leaving the filter paper. The pre-exposed male of *B. germanica* was gently placed in the centre of the filter paper and the time spent by the insect in each zone was monitored during 300 s using a digital video camera connected to a monitor (Sony, Tokyo, Japan).

The results were expressed as a Repellency Coefficient (RC = (Total Experimental Time − Time in Zone II)/Total Experimental Time). RC values vary between 0 (maximum attraction) and 1 (maximum repellency). RC = 0.5 indicates that the insect spent the same time in both zones (random distribution). To ensure random distribution on the arena, control experiments were performed using an arena where both halves were treated only with acetone. Nine independent replicates were performed for each bioassay.

To determine the role of NO in the behavioural response of the repellent DEET, insects were treated with SNAC before testing repellency. A solution of SNAC 40 mM was topically applied to cockroaches’ antennae using a microsyringe provided with a dispenser (Hamilton, Reno, NV, USA). Each insect received 1 µl/antenna. Immediately after treatment, repellency was tested as previously described. The concentration of SNAC to be tested was determined in preliminary experiments. Control groups received 1 µl/antenna of the vehicle of the solution (Triton X-100 1% in water) before testing repellency. A total of 17 independent replicates were performed.

### Electroantennographic bioassays

The electrical activity of cockroaches’ antennae in response to DEET was determined using the EAG technique. One *B. germanica* male was placed with its dorsal side up on a plasticine support. The animal was immobilized with thin copper wires that held the body all along. Glass electrodes filled with physiological solution (Ringer NaCl for insects, prepared in our laboratory) were connected to probes via Ag/AgCl filaments. The distal tip of the flagellum of one of the antennae was cut to ensure the contact of the haemolymph with the saline solution, and then was inserted into the recording electrode; the reference electrode was punctured to the base of the antenna. The remaining antenna was held with copper wires to avoid noise caused by eventual movements and it was not used for recording. Probes were connected to a signal acquisition interface (IDAC-2; Syntech, Stilwell, KS, USA) and the signals were recorded and analyzed with the aid of a software (EAG Pro; Syntech, Stilwell, KS, USA). The stimulus was delivered using a 3 s air puff which passed across a pipette containing a rectangular filter paper (2 cm × 1 cm) loaded with 25 µl of pure DEET. Three consecutive recordings were performed. Each presentation of the repellent was separated by a 60 s interval. The same procedure was followed in controls: a 3 s air puff passed across the pipette containing a clean filter paper; three consecutive recordings were performed. Each presentation of a clean air puff was separated by a 60 s interval.

This protocol was standardized in preliminary experiments. Nine independent replicates were performed. Each replicate was the mean of the three consecutive recordings performed in a single antenna. Each antenna was used only once.

**Figure 1:**
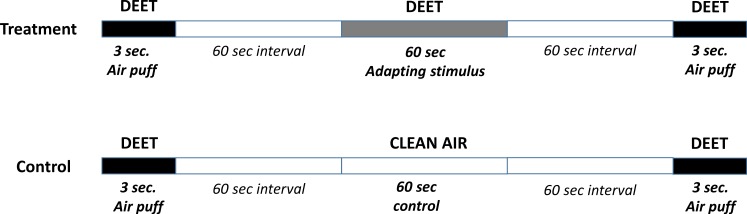
Adaptation protocol. Timeline of the protocol used for the presentation of the stimuli in order to reach sensory adaptation of the cockroaches’ antennae in response to a high stimulation with DEET.

For the adaptation experiments, the connected antenna was exposed to a long pulse of DEET of 60 s. The response of the antenna to DEET was registered as described above, before and after the exposure to the long pulse of DEET. In control groups, the responses of the antenna to DEET before and after the exposure to a 60 s. pulse of clean air were measured. The timeline of the presentation of the stimuli is presented in Fig. 1.

Ratios between the EAG amplitude after and before treatment were calculated in each experimental series (EAG after/EAG before). 10 independent replicates were performed in this series. Each replicate was the mean of three consecutive measurements performed in a single antenna. Each antenna was used only once.

To explore whether the NO/cGMP system participates in the signaling pathway of the adaptation phenomenon, pharmacological experiments were performed. Insects were treated with the NO donor SNAC (40 mM in distilled water + Triton X-100 1%) or the nucleotide analogue dbcGMP (2 mM in distilled water + Triton X-100 1%). SNAC releases NO in the internal compartment of the insect body. NO is a gaseous molecule, that diffuses inside the cells to elicit its effect. In the same way, the cyclic nucleotide analogues such as dbcGMP, can trespass the cell membrane and trigger the specific signaling cascade.

An amount of 1 µl of each substance was topically applied to the connected antennae using a microsyringe provided with a dispenser (Hamilton, Reno, NV, USA). Control groups received the same volume of solvent (distilled water + Triton X-100 1%). The response to DEET was registered before and after the application of each substance. Ratios between the amplitude of the electrical signal after and before treatment were calculated (EAG after/EAG before). A total of 6–10 independent replicates were performed. Each replicate was the mean of three consecutive measurements performed on a single antenna. Each antenna was used only once.

### Statistical analysis

In the behavioural experiments, RCs were compared using one way ANOVA, followed by Tukey’s test for *post hoc* comparisons when required. In the EAG bioassays, ratios of the signals before and after treatments were compared against the ratios of the respective controls using Student’s *t* test and *t* test for a parameter. When it was necessary a transformation of the data were preformed to meet the assumptions of the ANOVA.

## Results

**Figure 2:**
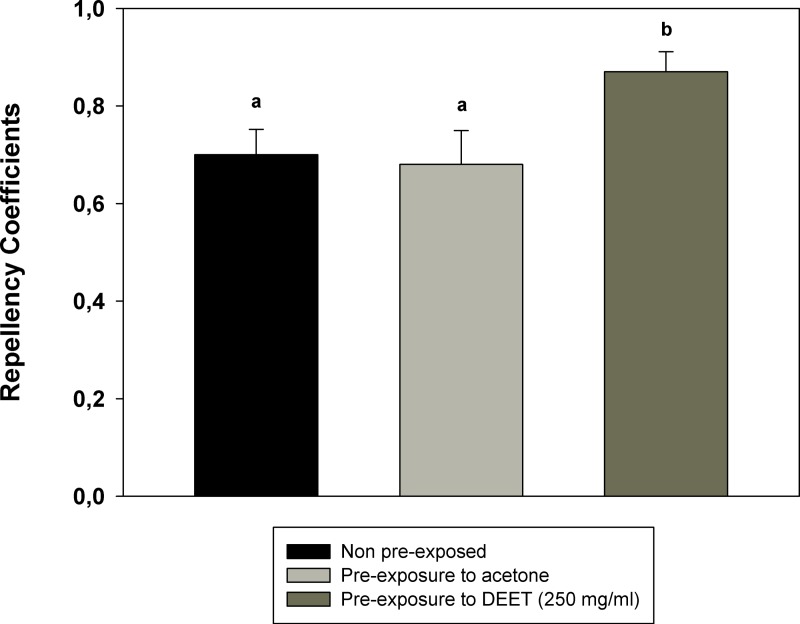
Repellency after previous exposure to DEET. Repellency Coefficients (RC) elicited by DEET after previous exposure of cockroaches to the same repellent. Two control groups are presented: the black bar indicates the mean repellency coefficient of insects with no previous treatment; the light grey bar indicates the mean repellency coefficient of insects pre-exposed only to acetone. Bars are the mean of the RCs of nine independent replicates. Error bars indicate standard error. Different letters indicates statistically significant differences (*p* < 0.05, one-way ANOVA and Tukey test for *post hoc* comparisons).

Figure 2 shows the RC of cockroaches pre-exposed to 250 mg/ml of DEET for 20 min. Repellency to 100 mg/ml of DEET was tested immediately after the end of the pre-exposure to the same substance. RCs of insects pre-exposed to DEET are significantly higher than RCs of non-pre-exposed or acetone-pre-exposed insects. That is, experience with DEET caused in *B. germanica* an increase in the repellency elicited by the same substance.

**Figure 3:**
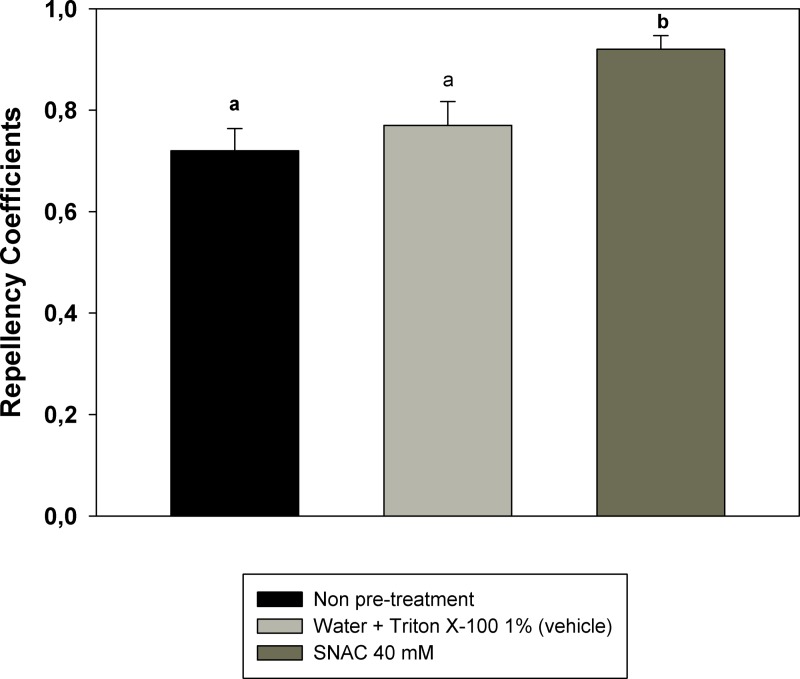
Modulation of DEET repellency by NO. RCs of cockroaches exposed to DEET after treatment with SNAC. Two control groups are presented: the black bar indicates the mean repellency coefficient of insects with no previous treatment; the light grey bar indicates the mean repellency coefficient of insects treated in the antennae with water + Tritón X-100 1% (vehicle). Bars are the mean of RCs of 17 independent replicates. Error bars indicate standard error. Different letters indicate statistically significant differences (*p* < 0.05; one-way ANOVA and Tukey test for *post hoc* comparisons).

In order to study whether NO participates in the modulation of the behaviour elicited by an odourant (DEET in this case), insects were topically treated with SNAC. Repellency elicited by DEET in treated cockroaches is shown in Fig. 3. RC of treated insects is higher than RCs of non-pre-treated or solvent-pre-treated insects. SNAC modulated the behavioural response to DEET in the same sense that occurred after previous exposure to the repellent.

We also measured the electrical activity of the antennae of *B. germanica* in response to DEET, to test if it is detected by cockroaches’ olfactory receptors. We measured a mean electrical response of 0.23 mV to the repellent.

**Figure 4:**
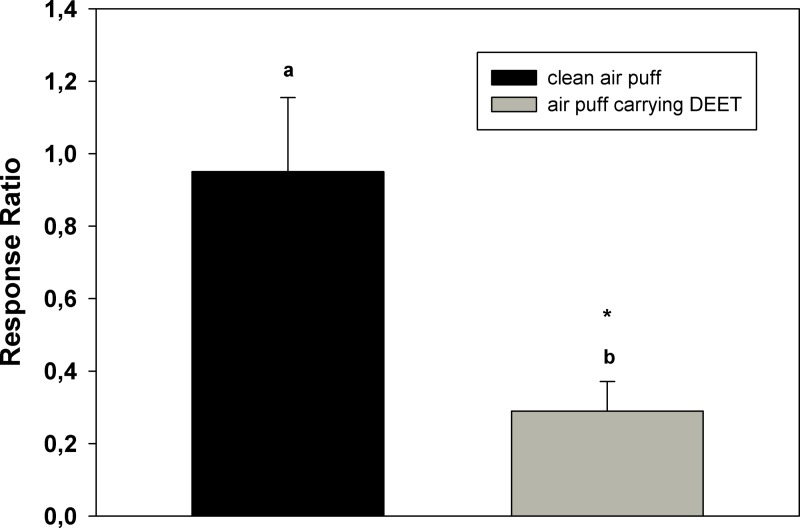
Adaptation of cockroaches’ antennae to DEET. Ratios of the EAG responses to DEET (EAG after/EAG before) after a long stimulation of the antenna preparation with the same substance. Bars are the mean of 10 independent replicates. Error bars indicate standard error. Different letters indicate statistically significant differences between ratios; the mark indicates significant difference from 1 (*p* < 0.05, Student’s *t* test and *t* test for a parameter respectively).

In order to test the occurrence of sensory adaptation, cockroaches’ antennae were exposed to a long pulse of DEET. Figure4 shows the ratio of the EAG responses after/before the long stimulation with DEET. The ratio of the responses after/before a clean air puff is near 1, while the ratio of the response after and before the pulse of DEET is lower than 1, indicating that the amplitude of the signal after stimulation was lower than the response to DEET before stimulation. The mean of the ratios obtained in the adaptation experiment is significantly different from control ratios.

**Figure 5:**
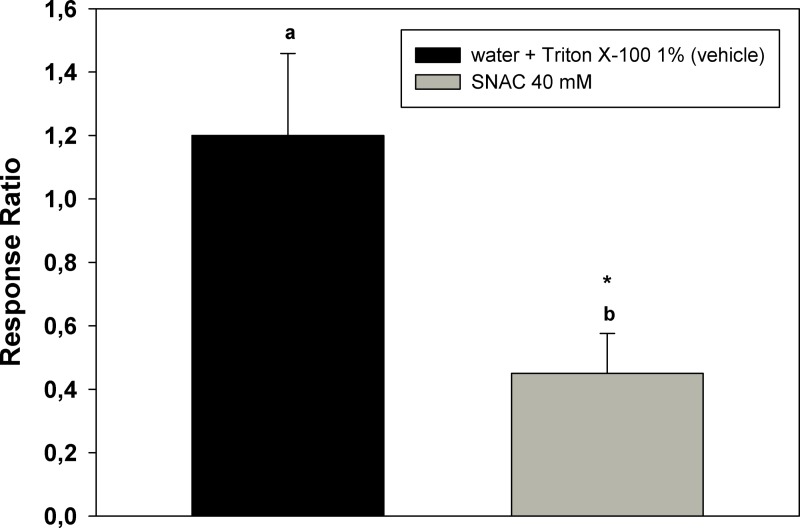
Modulation of the electrical response to DEET by NO. Ratios of the EAG responses to DEET (EAG after/EAG before) after treatment of the antenna preparation with SNAC. Bars indicate the mean of nine independent replicates. Error bars indicate standard errors. Different letters indicate statistically significant differences between ratios and the mark indicates significant difference from 1 (*p* < 0.05, Student’s *t* test; *t* test for a parameter respectively).

Figure 5 shows the ratio of the EAG responses to DEET after/before topical application of SNAC. The ratio of the signals from treated insects is lower than 1 and significantly different from the control ratio (*p* < 0.05).

**Figure 6:**
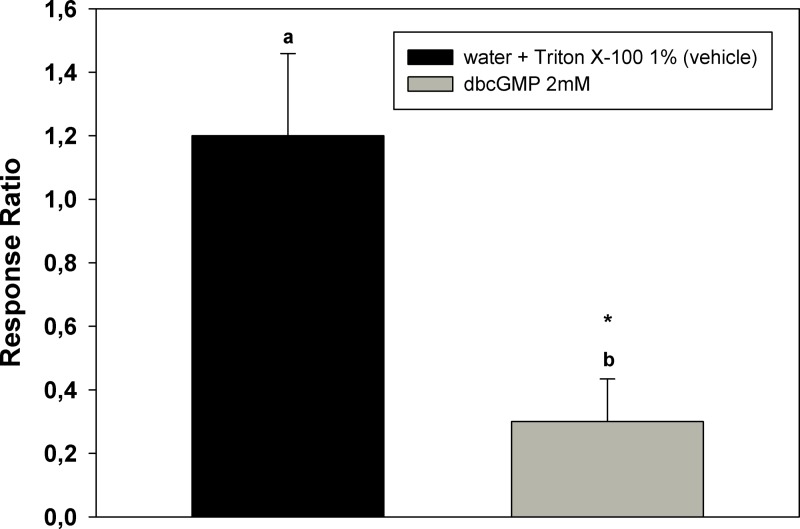
Modulation of the electrical response to DEET by cGMP. Ratios of the EAG responses to DEET (EAG after/EAG before) after treatment of the antenna preparation with dbcGMP. Bars indicate the mean of 6–9 independent replicates. Error bars indicate standard errors. Different letters indicate statistically significant differences between ratios and the mark indicates significant difference from 1 (*p* < 0.05, Student’s *t* test; *t* test for a parameter respectively).

A similar result was obtained when dbcGMP was topically applied to insects’ antennae. The ratio of the EAG signals after and before treatment with dbcGMP is lower than 1 and significantly different from control ratio (*p* < 0.05). These results are shown in Fig. 6.

Figures 5 and 6 shows that the chemical treatment of the antennae caused a decrease in the response to DEET similar of that observed after the sustained stimulation of the antennae with DEET, showed in Fig. 4, suggesting a role of NO/cGMP system in the adaptation phenomenon.

## Discussion

In this work we studied the effect of pre-exposure to the insect repellent DEET on the behavioural and electrical response of male cockroaches to the same substance. We showed that previous exposure to the repellent caused an increase in the repellency. Moreover, pre-treatment with NO donor SNAC also increased repellency.

We also measured the electrical activity of the antennae in response to DEET, showing that cockroaches are able to smell the repellent. In addition, we showed that the exposure to a long pulse of DEET reduced the amplitude of the electrical response of cockroaches’ antennae elicited by the same compound, in the same way that occurred after treating the antennae with SNAC or dbcGMP.

Different neuronal processes occurring both at the peripheral and central level can explain changes in responsiveness to chemical stimuli. Sensory adaptation implies the reduction of sensitivity of receptors neurons after a sustained stimulation.

The exposure to high concentrations of an odour stimulus may elicit sensory adaptation in the olfactory receptor neurons (ORNs). This phenomenon occurs in almost all sensory cells and consists of the regulation of the sensitivity of the sensory system to different stimulus intensities (Zufall & Leinders-Zufall, 2000) to allow the retention of high sensitivity during continuous or repetitive exposure to stimuli. Adaptation is associated with a shift in the stimulus–response curve to higher concentrations (Borroni & Atema, 1988).

In the case of olfaction, there are some reports of reduced responsiveness in insects pre-exposed to chemicals. Partial or total loss in responsiveness after experience with chemical stimuli was reported in *D. melanogaster* (Cobb & Domain, 2000; Boyle & Cobb, 2005). The response of larvae to certain attractants decreased after pre-exposure to the same substances. These authors interpreted these results in terms of sensory adaptation.

We observed in previous works that the repellency elicited by DEET, menthyl acetate and geraniol decreased after previous exposure to the same substances, in nymphs of the blood-sucking bug *R. prolixus*. This effect was reversible and dose-dependent (Sfara et al., 2011; Lutz, Sfara & Alzogaray, 2014). Similar results were obtained by Stanczyk et al. (2013), who observed a decrease of the behavioural and electrical response to DEET in mosquitoes of the species *Ae. aegypti*, after previous exposure to the repellent.

However, previous experience with an odour not always causes a decrease in the sensitivity of sensory neurons and in the behavioural response associated. In this work we found that cockroaches previously exposed for 20 min to a high concentration of DEET, showed more repellency when tested with the same compound, compared with non pre-exposed insects. This result indicates that, in the case of cockroaches, previous experience with DEET enhanced the repellency elicited by the same compound. Anderson et al. (2007) reported that males of the noctuid moth *Spodoptera littoralis* were more attracted by the specific sex pheromone after previous exposure to it. These authors also observed an increase in the sensitivity of neurons of the antennal lobe of pre-exposed males, indicating that the change observed in the behaviour was associated to a neuronal change at the central level.

The decrease in the behavioural response of an insect towards a chemical stimulus may also be associated to changes in the neuronal activity at the central level. It has been observed that *Drosophila* exposed for four days to odours exhibited a change in the activity of the glomeruli in an odour-selective manner, although the electrical response of the antennae remained normal (Devaud, Acebes & Ferrús, 2001).

In this work we found that treatment of the cockroaches’ antennae with the NO donor SNAC caused an increase in the repellency elicited by DEET, in the same way that occurs after previous exposure to the same substance. In these series of experiments, the chemical treatment mimicked the effect of the long stimulation, suggesting that NO plays a role in the modulation of the repellency behaviour, possibly at the sensory level.

The role of NO/cGMP in the modulation of the behavioural response towards repellents was studied also in the blood-sucking bug *R prolixus*. When SNAC or dbcGMP were applied to the bug’s antennae, a decrease of the repellency elicited by DEET, menthyl acetate or geraniol was observed (Sfara, Zerba & Alzogaray, 2008; Lutz, Sfara & Alzogaray, 2014). A similar decrease of repellency occurred after previous exposure to high concentrations of the same repellents. In all cases, the behavioural response was recovered a few minutes after treatment, suggesting a peripheral phenomenon (Sfara et al., 2011; Lutz, Sfara & Alzogaray, 2014).

In the case of pre-exposure to DEET and natural repellents produced a decrease in the behavioural response to the same substances. Conversely, pre-exposure of cockroaches to DEET increased the repellency. In both cases, the modulation of the response to repellents elicited by treatment with SNAC occurs in the same direction that the modulation caused by the previous exposure. These results suggest that NO participates in this process, regardless of the direction in which behavioural change occurs.

In addition, we recorded the electrical activity of the cockroaches’ antennae in response to DEET. We observed a measurable and reproducible signal in response to DEET, indicating that cockroaches smell it. This result is in agreement with the observations made in *Ae. aegypti* and *Culex quinquefasciatus* mosquitoes, in which authors measured the electrical response to DEET in the whole antenna and in a single sensillum (Syed & Leal, 2008; Stanczyk et al., 2013). These results confirm that DEET acts as an odour stimulus for a number of insect species, including a non-haematophagous one, such as *B. germanica*.

We also showed that the exposure of *B. germanica* antennae to a long pulse of DEET produced a decrease in the electrical response to the same substance. A total recovery of the response was observed 10 min after exposure. These results suggest that a sensory adaptation phenomenon occurred as a consequence of a long stimulation of the antennae with an odour of high concentration, such as DEET.

This result was expected because sensory adaptation is a phenomenon that occurs in sensory neurons, after the exposure to a stimulus of high intensity. In order to study a possible role of NO in the adaptation phenomenon to DEET observed in *B. germanica*, antennae were topically treated with the NO donor SNAC. After treatment, the response of the antennae to DEET decreased significantly. This reduction was similar to the reduction occurred when the antennae were exposed to the repellent itself. The response to DEET was recovered a few minutes after SNAC application, indicating a peripheral and transient effect of the substance. A similar result was obtained when antennae were treated with dbcGMP, suggesting the participation of both NO/cGMP in the transduction pathway of this phenomenon.

These results are in agreement with observations made by other authors. Redkozubov (2000) observed a decrease in the receptor current of the sensilla of *Bombix mori* in response to pheromone components, when the receptor was treated with dbcGMP.

Newland & Yates (2007) showed that the chemical treatment with NO donors of the salt receptor of the ovipositor in the locust *S. gregaria*, modulated its electrical activity. The salt receptor senses the concentration of NaCl present in the substrate where the female locust oviposits and regulates the digging rhythm of the ovipositor valves. The intracellular synthesis of NO decreases the electrical activity of the NaCl receptor and as a consequence the oviposition rhythm also decreases.

In summary, in this study we showed that the exposure to a chemical stimulus may modify the behaviour associated to that stimulus, either by increasing as well as decreasing the behavioural response. We found that NO participates in this phenomenon. We also showed that insects’ antennae perceive DEET as an odour. A long exposure of the antennae to DEET caused a transient decrease in the response of the antennae to the same compound. This was also observed when SNAC or dbcGMP were applied on the antennae. A limitation of this study is the possible unspecific effects of N-acetylcysteine, used to synthesize SNAC, or cGMP. These substances were not directly tested on cockroaches’ antennae as additional controls and this point has to be considered for future experiments. However, the observations made in this work can still be related with specific intracellular effects of NO and cGMP, because they are in consonance with other published results in which specific effects of NO/cGMP system were described in different neuronal systems, using SNAC as NO donor and dbcGMP as cGMP analogue. Therefore we suggest a role of NO/cGMP system in the modulation of peripheral responses of neurons in this species.

We propose a model for these observations: sensory adaptation of the antennae of *B. germanica* that occurs after a long exposure to DEET is mediated by NO/cGMP and caused a general sensitization of contact chemoreceptors. In this way, when olfactory-adapted insects where exposed to DEET-treated surface, a higher repellency was observed (at the behavioural level) as a consequence of an increased sensitivity of contact chemoreceptors. Clearly this is just an assumption, and more experiments are required to confirm the hypothesis.

## Conclusions

In this work we showed that the previous exposure to the repellent DEET enhanced the response of male cockroaches to the same substance. In a similar way, treatment with the NO donor SNAC increased the response to DEET of treated cockroaches.

We also found that cockroaches’ antennae perceive DEET as an odorant molecule. We recorded the electrical activity of the antennae of *B. germanica* in response to the repellent, and we observed that the response decreased after a long exposure to the substance. In the same way, the electrical response to DEET decreased after treatment of the cockroaches’ antennae with the nitric oxide donor SNAC or the cyclic nucleotide analogue dbcGMP, suggesting a role of these molecules in the transduction pathway of the sensory adaptation phenomenon.

##  Supplemental Information

10.7717/peerj.2150/supp-1 Data S1 Sfara et al. raw-data

## Additional Information and Declarations

### Data Availability

The following information was supplied regarding data availability: The raw data has been supplied as Data S1.
