# Supplementary material for: Modulation of the behavioral and electrical responses to the repellent DEET elicited by the pre-exposure to the same compound in Blattella germanica
Source: PeerJ. 2016 Jun 28;4:e2150. doi: 10.7717/peerj.2150 (PMC4928467; doi:10.7717/peerj.2150)
Supplement: Data S1 [file peerj-04-2150-s001.docx]

| **Repellence elicited by DEET after previous exposure of the cockroaches to the same repellent (Fig 2).** | | | | | | |  |  |
| --- | --- | --- | --- | --- | --- | --- | --- | --- |
| **Repellence Coefficients (RC)** | | | |  | |  |  |  |
| **Replicates** | | **RC non pre-exposed insects** | | **RC acetone pre-exposed insects** | | **RC DEET pre-exposed insects** |  |  |
| 1 | | 0,8 | | 0,71 | | 0,7 |  |  |
| 2 | | 0,75 | | 0,89 | | 0,99 |  |  |
| 3 | | 0,63 | | 0,45 | | 1 |  |  |
| 4 | | 0,51 | | 0,66 | | 0,73 |  |  |
| 5 | | 0,9 | | 0,61 | | 0,78 |  |  |
| 6 | | 0,75 | | 0,94 | | 0,98 |  |  |
| 7 | | 0,43 | | 0,95 | | 0,78 |  |  |
| 8 | | 0,86 | | 0,52 | | 0,98 |  |  |
| 9 | | 0,67 | | 0,40 | | 0,92 |  |  |
| **Mean** | | **0,70** | | **0,68** | | **0,87** |  |  |
|  | |  | |  | |  |  |  |
|  | |  | |  | |  |  |  |
|  | |  | |  | |  |  |  |
| Insects were pre-exposed to 250 mg/ml of DEET vapours, | | | | | |  |  |  |
| and then repellency was tested using a solution of 100 mg/ml of the same substance. | | | | | | |  |  |
|  |  | | |  | |  |  |  |
|  |  | | |  | |  |  |  |
| **Repellence elicited by DEET after treatment of the cockroaches´ antennae with the NO donor SNAC (Fig 3).** | | | | | | |  |  |
|  |  | | |  | |  |  |  |
| **Replicates** | **RC non pre-treated insects** | | | **RC solvent pre-treated insects** | | **RC SNAC pre-treated insects** |  |  |
| 1 | 0,41 | | | 0,77 | | 0,84 |  |  |
| 2 | 1 | | | 0,72 | | 0,82 |  |  |
| 3 | 0,56 | | | 0,83 | | 0,97 |  |  |
| 4 | 0,91 | | | 0,84 | | 0,79 |  |  |
| 5 | 1 | | | 1 | | 1 |  |  |
| 6 | 0,75 | | | 0,44 | | 0,95 |  |  |
| 7 | 0,8 | | | 0,75 | | 1 |  |  |
| 8 | 0,86 | | | 0,69 | | 0,98 |  |  |
| 9 | 0,43 | | | 0,2 | | 1 |  |  |
| 10 | 0,67 | | | 0,79 | | 0,94 |  |  |
| 11 | 0,75 | | | 0,8 | | 0,97 |  |  |
| 12 | 0,68 | | | 0,89 | | 0,99 |  |  |
| 13 | 0,67 | | | 0,88 | | 0,97 |  |  |
| 14 | 0,9 | | | 0,9 | | 0,99 |  |  |
| 15 | 0,63 | | | 0,82 | | 1 |  |  |
| 16 | 0,75 | | | 0,98 | | 0,72 |  |  |
| 17 | 0,51 | | | 0,7 | | 0,65 |  |  |
| **Mean** | **0,72** | | | **0,77** | | **0,92** |  |  |
| Insects were treated with 1 µl of NO donor SNAC (40mM) applied on the antennae; | | | | | |  |  |  |
| control groups were non-pretreated or pre-treated with 1 µl of distilled water + Triton X-100 (solvent). | | | | | | |  |  |
| Then, repellency was tested with 100 mg/ml DEET | | | | | |  |  |  |
|  |  | | |  | |  |  |  |
| **Amplitude of the electrical signal of the antennae of cockroaches in response to DEET (Result showed in the text)** | | | | | | |  |  |
|  |  | | |  | |  |  |  |
| **Replicate** | **Amplitude (in mV)** | | |  | |  |  |  |
| 1 | 0,18 | | |  | |  |  |  |
| 2 | 0,19 | | |  | |  |  |  |
| 3 | 0,46 | | |  | |  |  |  |
| 4 | 0,17 | | |  | |  |  |  |
| 5 | 0,16 | | |  | |  |  |  |
| 6 | 0,18 | | |  | |  |  |  |
| 7 | 0,3 | | |  | |  |  |  |
| 8 | 0,28 | | |  | |  |  |  |
| 9 | 0,14 | | |  | |  |  |  |
| **Mean** | **0,23** | | |  | |  |  |  |
| Each replicate is the mean of three consecutive measurements on a single antenna | | | | | | |  |  |
|  |  | | |  | |  |  |  |
|  |  | | |  | |  |  |  |
| **Electrical response of the antennae to DEET after a long stimulation with the same substance (Fig 4).** | | | | | | |  |  |
| **Control group: adapting stimulus: clean air** | | | | | |  |  |  |
| **Replicate** | **Amplitude before adaptation** | | | **Amplitude after adaptation** | | **Ratio (after/before)** |  |  |
| 1 | 0,06 | | | 0,01 | | 0,17 |  |  |
| 2 | 0,76 | | | 0,62 | | 0,82 |  |  |
| 3 | 0,19 | | | 0,17 | | 0,89 |  |  |
| 4 | 0,28 | | | 0,32 | | 1,1 |  |  |
| 5 | 0,02 | | | 0,04 | | 2 |  |  |
| 6 | 1,2 | | | 1,8 | | 1,5 |  |  |
| 7 | 0,17 | | | 0,02 | | 0,12 |  |  |
| 8 | 0,19 | | | 0,18 | | 0,95 |  |  |
| 9 | 0,13 | | | 0,22 | | 1,7 |  |  |
| 10 | 0,08 | | | 0,02 | | 0,25 |  |  |
| **Mean** |  | | |  | | **0,95** |  |  |
|  |  | | |  | |  |  |  |
|  |  | | |  | |  |  |  |
|  |  | | |  | |  |  |  |
| **Experimental group: adapting stimulus: DEET** | | | | | |  |  |  |
| **Replicate** | **Amplitude before adaptation** | | | **Amplitude after adaptation** | | **Ratio (after/before)** |  |  |
| 1 | 0,20 | | | 0,02 | | 0,10 |  |  |
| 2 | 0,16 | | | 0,08 | | 0,5 |  |  |
| 3 | 0,03 | | | 0 | | 0 |  |  |
| 4 | 0,36 | | | 0,11 | | 0,31 |  |  |
| 5 | 0,76 | | | 0,48 | | 0,63 |  |  |
| 6 | 0,1 | | | 0 | | 0 |  |  |
| 7 | 0,13 | | | 0,04 | | 0,31 |  |  |
| 8 | 0,21 | | | 0,05 | | 0,24 |  |  |
| 9 | 0,43 | | | 0,02 | | 0,05 |  |  |
| 10 | 0,21 | | | 0,15 | | 0,71 |  |  |
| **Mean** |  | | |  | | **0,29** |  |  |
|  |  | | |  | |  |  |  |
| **Electrical response of the antennae to DEET after treatment with SNAC (Fig 5) and dbcGMP (Fig 6).** | | | | | | |  |  |
| **Control group: application of solvent (distilled water + Triton X-100 1%)** | | |  | |  |  |  |  |
|  | | |  | |  |  |  |  |
| **Replicate** | | | **Amplitude before treatment** | | **Amplitude after treatment** | **Ratio (after/before)** |  |  |
| 1 | | | 0,25 | | 0,11 | 0,45 |  |  |
| 2 | | | 0,5 | | 0,62 | 1,2 |  |  |
| 3 | | | 0,34 | | 0,41 | 1,2 |  |  |
| 4 | | | 0,95 | | 0,67 | 0,7 |  |  |
| 5 | | | 0,1 | | 0,26 | 2,6 |  |  |
| 6 | | | 0,55 | | 0,64 | 1,2 |  |  |
| 7 | | | 0,97 | | 0,98 | 1 |  |  |
| **Mean** | | |  | |  | **1,2** |  |  |
|  | | |  | |  |  |  |  |
|  | | |  | |  |  |  |  |
| **SNAC** | | | | | |  |  |  |
| **Replicate** | **Amplitude before treatment** | | | **Amplitude after treatment** | | **Ratio (after/before)** |  |  |
| 1 | 0,33 | | | 0,095 | | 0,29 |  |  |
| 2 | 0,1 | | | 0,03 | | 0,3 |  |  |
| 3 | 0,55 | | | 0,16 | | 0,29 |  |  |
| 4 | 0,15 | | | 0,11 | | 0,73 |  |  |
| 5 | 0,25 | | | 0,017 | | 0,07 |  |  |
| 6 | 0,11 | | | 0,09 | | 0,82 |  |  |
| 7 | 0,26 | | | 0,03 | | 0,11 |  |  |
| 8 | 0,36 | | | 0,1 | | 0,28 |  |  |
| 9 | 0,20 | | | 0,24 | | 1,2 |  |  |
| **Mean** |  | | |  | | **0,45** |  |  |
|  |  | | |  | |  |  |  |
| **dbcGMP** |  | | |  | |  |  |  |
| **Replicate** | **Amplitude before treatment** | | | **Amplitude after treatment** | | **Ratio (after/before)** |  |  |
| 1 | 0,14 | | | 0,02 | | 0,11 |  |  |
| 2 | 1,32 | | | 0 | | 0 |  |  |
| 3 | 0,07 | | | 0,06 | | 0,8 |  |  |
| 4 | 0,14 | | | 0,08 | | 0,57 |  |  |
| 5 | 0,19 | | | 0 | | 0 |  |  |
| 6 | 0,37 | | | 0,12 | | 0,32 |  |  |
| **Mean** |  | | |  | | **0,3** |  |  |
|  |  | | |  | |  |  |  |
| In all cases, each replicate is the mean of three consecutive measurements on a single antenna |  | | |  | |  |  |  |
|  |  | | |  | |  |  |  |
|  |  | | |  | |  |  |  |
|  |  | | |  | |  |  |  |
|  |  | | |  | |  |  |  |
|  |  | | |  | |  |  |  |
|  |  | | |  | |  |  |  |
|  |  | | |  | |  |  |  |
|  |  | | |  | |  |  |  |
|  |  | | |  | |  |  |  |
|  |  | | |  | |  |  |  |
|  |  | | |  | |  |  |  |
|  | | | | | | |  |  |
